# Acute kidney injury due to hemoglobin cast nephropathy secondary to autoimmune hemolytic anemia: a case report

**DOI:** 10.3389/fmed.2026.1898126

**Published:** 2026-10-07

**Authors:** Xiaoqian Qian, Jun Zou

**Affiliations:** Renal Division, Department of Internal Medicine, Xin Hua Hospital Affiliated to Shanghai Jiao Tong University School of Medicine, Shanghai, China

**Keywords:** acute kidney injury, autoimmune hemolytic anemia, case report, direct antiglobulin test (DAT), hemoglobin cast nephropathy

## Abstract

**Background:**

Autoimmune hemolytic anemia (AIHA) is an uncommon acquired disorder that can lead to life-threatening intravascular hemolysis and acute kidney injury (AKI). Hemoglobin cast nephropathy is a rare but severe complication of massive hemolysis, often underrecognized in clinical practice.

**Case presentations:**

We describe a 57-year-old female with no prior chronic kidney disease who presented with nausea, vomiting, oliguria, edema, and rapidly progressive renal dysfunction after a presumed preceding infectious-like episode. Laboratory investigations revealed severe anemia, elevated hemolytic markers, and a positive direct antiglobulin test (DAT) for complement C3 (C3d) and negative for IgG. Renal biopsy demonstrated acute tubular injury with hemoglobin casts confirmed by immunostaining, consistent with hemoglobin cast nephropathy. The patient was treated with intermittent renal replacement therapy, intravenous methylprednisolone, and supportive care. Renal function and hemoglobin levels gradually normalized, and she remained well without relapse over 11.5 months of follow-up.

**Conclusion:**

This case highlights that when acute kidney injury and hemolytic anemia occur concurrently, hemoglobin cast nephropathy should be strongly suspected. Renal biopsy is valuable when the diagnosis is uncertain. Early immunosuppressive therapy, renal replacement therapy, and supportive care can lead to favorable outcomes.

## Introduction

Autoimmune hemolytic anemia (AIHA) is caused by the destruction of an individual's own red blood cells by autoantibodies directed against red-cell antigens (1, 2), leading to hemolysis and hemolytic anemia. Red-cell contents are relased into the circulation, giving rise to the characteristic clinical and biochemical features (3). AIHA is a serious and potentially life-threatening condition typically diagnosed on the basis of hemolysis markers and a positive Direct Antiglobulin Test (DAT). AIHA is classified as either primary (idiopathic) or secondary according to the thermal optimum of the autoantibody into warm-antibody AIHA (wAIHA), cold-antibody AIHA (including cold agglutinin disease/syndrome and paroxysmal cold hemoglobinuria), mixed-type AIHA, and DAT-negative AIHA (1, 4). Prime AIHA accounts for more than 60% of cases, whereas secondary AIHA may be caused by autoimmune diseases, hematological malignancies, and infections induced by pathogens such as Epstein–Barr virus (EBV), cytomegalovirus (CMV), and Mycoplasma pneumoniae.

Intravascular hemolysis is a pathological process-not an independent disease entity-characterized by the destruction of red blood cells with the circulation, with release of free hemoglobin into the plasmas (5). When massive, free hemoglobin can overwhelm its binding partner haptoglobin, pass the glomerular filtration barrier, and precipitate within the renal tubules, causing acute kidney injury through hemoglobin cast nephropathy. In this case report, we describe a patient who developed complement-positive autoimmune hemolytic anemia following a presumed infectious-like episode, leading to oliguric acute renal injury within a short period. The patient recovered and was discharged after treatment.

## Case presentations

A 57-year-old woman was admitted to the renal division on August 9, 2025, with a 10-day history of progressively worsening nausea and vomiting accompanied by watery diarrhea (3–4 times daily), edema and oliguria. She has no known chronic comorbidities and no prior history of kidney disease. Ten days before admission (July 31, 2025), after exposure to rain, she developed nausea, vomiting, and diarrhea with myalgia; she self-reported a subjective sensation of fever, though body temperature was not measured during the pre-admission illness, and she had no respiratory symptoms. She also reported a single episode of mildly bloody urine with a small blood clot (not tea- or cola-colored). She denied use of illicit drugs, tobacco, or alcohol.

No prior creatinine measurement was available and the patient had no history of kidney disease; the “baseline” creatinine of 92 μmol/L and “baseline” hemoglobin of 115 g/L cited in the original figures represent the upper limit of the institutional creatinine reference range (46–92 μmol/L) an the lower limit of the hemoglobin reference range (115–150 g/L), respectively, used as surrogates for a normal preillness baseline. A routine check on March 9, 2025 (approximately five months before admission) showed a hemoglobin of 123 g/L with normal white-cell (4.53 × 10^9^/L) and platelet (143 × 10^9^/L) counts, supporting a normal pre-illness baseline.

### Outpatient course (one week before admission)

On August 1, 2025, a community hospital evaluation showed hemoglobin 80 g/L, lactate dehydrogenase (LDH) 1627 U/L, total bilirubin 50.8 μmol/L, creatinine 426.6 μmol/L, blood urea nitrogen (BUN) 27.7 mmol/L, and D-dimer 18.25 mg/L, with urinalysis positive for occult blood (2+). On August 2, 2025, a municipal hospital evaluation showed hemoglobin 77 g/L, creatinine 456 μmol/L, reticulocytes 2.76%, and total bilirubin 25.8 μmol/L, with 24 h urine protein of 768 mg. The patient received antihypertensive and iron supplementation therapy, followed by ceftazidime and low-molecular-weight heparin for approximately one week, but edema worsened and urine output declined.

### On admission (day 1)

Physical examination revealed hypertension (167/90 mmHg), moderate edema, tachycardia (110 beats/min), and coarse breath sounds. Laboratory findings included hemoglobin 64 g/L, red blood cells 1.99 × 10^12^/L, white blood cells 9.52 × 10^9^/L, platelets 213 × 10^9^/L, creatinine 807 μmol/L, blood urea nitrogen 48.5 mmol/L, LDH 352 U/L, total bilirubin 9.4 μmol/L, C-reactive protein 17 mg/L, and procalcitonin 0.71 ng/mL. Urinalysis showed protein 500 mg/dL, hemoglobin (dipstick) 250/*μ*L, 381 erythrocytes/μL, and 81 leukocytes/μL; the 24 h urine protein was 1,427.86 mg (urine volume 650 mL). A urine red-cell dysmorphism study showed 47,500 red cells/mL reported as non-glomerular. A summary of key laboratory values is provided in Table 1. Immunohematological and secondary-cause workup: The direct antiglobulin test (DAT) was positive for complement C3 (C3d) and negative for IgG; the indirect antiglobulin test and antibody screening were both negative.

**Table 1:** Patient's diagnostic parameters after admission with key findings highted.

Category	Parameters	Value, units	Reference range
Serum-CRP and blood routine test	Red blood cell	1.99 * 10^12^/L↓	3.8–5.1 * 10^12^/L
	Hemoglobin	64 g/L↓	115–150 g/L
	White blood cell	9.52 * 10^9^/L↑	3.5–9.5 * 10^9^/L
	Platelets	213 * 10^9^/L	125–300 * 10^9^/L
	CRP	17 mg/L↑	<8 mg/L
	Reticulocyte percentage	9.23%↑	0.5–1.5%
Serum-liver function	ALT	14 U/L	<35 U/L
	AST	34 U/L	14–16 U/L
	TBIL	9.4 μmol/L	3–22 μmol/L
	DBIL	0 μmol/L	0–5 μmol/L
Serum-electrolytes and renal function	Serum sodium	128 mmol/L↓	137–145 mmol/L
	Serum potassium	3.6 mmol/L	3.5–5.1 mmol/L
	Serum phosphate	2 mmol/L↑	0.81–1.45 mmol/L
	Serum calcium	2.15 mmol/L	2.1–2.55 mmol/L
	Serum creatinine	807 µmol/L↑	46–92 µmol/L
	Serum urea nitrogen	48.5 mmol/L↑	2.5–6.1 mmol/L
Serum-serology	ANA	(−)	(−)
	Anti-dsDNA	<10 IU/mL	<100 IU/mL
	Anti-PR3	<2 RU/mL	<20 RU/mL
	Anti-MPO	<2 RU/mL	<20 RU/mL
	Anti-GBM	<2 RU/mL	<20 RU/mL
	Complement C3	0.85 g/L↓	0.9–1.8 g/L
	Complement C4	0.2 g/L	0.1–0.4 g/L
Direct antiglobulin test	Direct antiglobulin test	Positive	Negative
	Anti-IgG	Negative	Negative
	Anti-C3	Positive	Negative
Haptoglobin assay	Haptoglobin	0.84 g/L	0.3–2 g/L
Serum-other	CK	36 U/L	40–200 U/L
	LDH	352 U/L↑	120–250 U/L
	Procalcitonin	0.71 ng/mL↑	<0.5 ng/mL
	D-dimer	2.2 mg/L FEU	0–0.5 mg/L FEU
Urine general analysis	urine specific gravity	1.013	1.010–1.030
	RBC	381.04/µL↑	<10
	WBC	81.18/µL↑	<12
	Protein/creatinine ratio	1,315.82 mg/g↑	<30
Urine red blood cell dysmorphism test	Total red blood cell count	47,500/mL↑	
	Final impression	Non-renal hematuria	
Urine-other	24 h urine protein	1,427.86 mg/24H↑	0–150 mg/24H
	24 h urine volume	650 mL	

Monospecific testing for IgA and IgM and eluate testing were not available in our institution. The cold agglutinin test (tube agglutination with serial dilution) was negative (titer < 1:32); thermal amplitude is not routinely reported in our laboratory. Antinuclear antibody, anti-dsDNA, anti-ENA, anti-CCP, rheumatoid factor, ANCA (anti-PR3 and anti-MPO), anti-GBM, and antiphospholipid antibodies were all negative. Complement C3 was mildly reduced (0.85 g/L; reference 0.9–1.8 g/L) with normal C4 (0.20 g/L). Serum protein electrophoresis, immunofixation, and serum free light chains showed no monoclonal gammopathy, and there was no clinical, laboratory, or imaging evidence of lymphoproliferative disease or malignancy.

Paroxysmal nocturnal hemoglobinuria was excluded by high-sensitivity flow cytometry (neutrophil FLAER/CD24, monocyte FLAER/CD14, and red-cell CD59 with type II [partial-deficiency] and type III [severe-deficiency] clones, all <0.1%), and glucose-6-phosphate dehydrogenase (G6PD) deficiency screening was normal. There was no history of exposure to drugs classically associated with hemolysis (rifampicin, oxidant agents, or herbal preparations), no recent surgery or mechanical cardiac device, and no transfusion history.

### Exclusion of thrombotic microangiopathy

Platelets were never below the lower limit of normal (trend: 213, 244,319, 255, 150, 150, 244 × 10^9^/L from day 1 to week 4). The peripheral blood smear was negative (no schistocytes, no spherocytes, and no red-cell agglutination). Coagulation studies were unremarkable. Stool Shiga-toxin testing and ADAMTS13 activity were not performed because the diarrhea had completely resolved and the platelet count was already normal by the time of admission one week after symptom onset. Together with the absence of thrombotic microangiopathy-associated lesions on renal biopsy, these findings made thrombotic microangiopathy clinically improbable.

Serial hemolysis parameters: LDH declined from 352 U/L (day 2) to 286 (day 7), 203 (day 14), and 201 U/L (day 18), falling below the upper limit of normal (250 U/L) by day 14. Indirect bilirubin was 9.4 μmol/L (day 1), 3.6 (day 7), 4.9 (day 14), and 5.4 μmol/L (day 18). Haptoglobin remained within the normal range (0.84 g/L on day 7; 1.06 g/L on day 18). Reticulocytes peaked at 9.23% on day 7 (absolute reticulocyte count 228 × 10^9^/L), declining to 7.59% (day 10), 2.6% (day 14), and 1.91% (day 18). Urine hemoglobin (dipstick) declined from 250/μL to negative during follow-up. Urine hemosiderin was not measured, but the renal biopsy showed no hemosiderin deposition within tubular epithelial cells.

## Therapeutic intervention

Renal replacement therapy was initiated in day 3 (August 11, 2025) primarily for oliguria complicated by volume-overload-induced heart failure refractory to diuretics, together with prominent uremic symptoms (marked anorexia, nausea, and vomiting) and metabolic acidosis (CO₂ combining power 16.0 mmol/L). A total of three low blood flow intermittent hemodialysis were delivered: 2 kg ultrafiltration over 3.5 h on August 11 (day 3), 3 kg ultrafiltration over 4 h on August 13 (day 5), and 2 kg over 2 h on August 15 (day 7). By day 10 (August 18), urine output had recovered to 1750mL with resolution of heart failure and uremic manifestations, and renal replacement therapy was discontinued.

Immunosuppression: intravenous methylprednisolone 40 mg daily (0.8 mg/kg/day for a 50 kg patient) was initiated on day 3 (August 11) and continued for 16 days, followed by 20 mg daily for 2 days, then conversion to oral methylprednisolone 12 mg/day, maintained for approximately 10 weeks (August 29 to November 7, 2025), then tapered stepwise (10 → 8 → 4 → 2 mg/day) and discontinued on January 30, 2026.

Renal biopsy (day 11, August 19, 2025) was performed because the hemolysis-related manifestations after admission were relatively mild, and acute kidney injury could not be confidently attribute to hemolysis alone; volume-depletion-associated ischemic injury and toxic-mediated AKI required exclusion. Light microscopy showed 14 glomeruli without global sclerosis, moderate mesangial matrix expansion in a few glomeruli, and extensive acute tubular injury (microvillus loss, tubular dilatation, vacuolar degeneration, and intraluminal granular, erythrocyte, and hemoglobin casts), with focal mild interstitial inflammation and focal tubular atrophy with interstitial fibrosis involving approximately 5% of the cortex. Immunofluorescence was negative for IgA, IgG, IgM, C1q, C3, and *κ* and *λ* light chains. Electron microscopy showed tubular epithelial vacuolar degeneration without electron-dense deposits. Hemoglobin immunostaining was positive in the majority of tubular casts (Figure 1); myoglobin immunostaining was negative, and no granular hemosiderin was observed within tubular epithelial cells (iron stain not performed).

**Figure 1:**
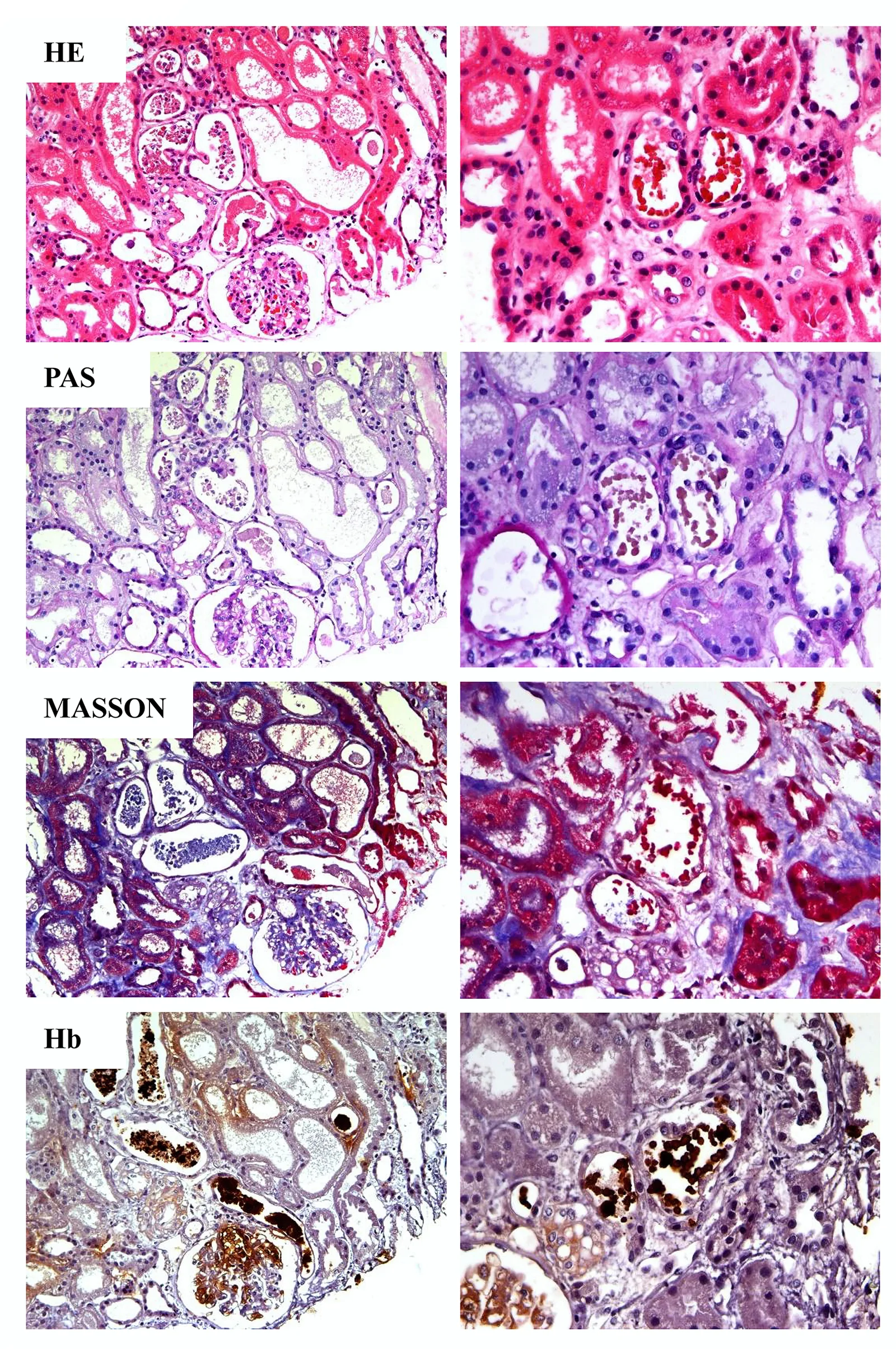
Kidney biopsy specimens. Granular casts in the tubular lumens were observed by Hematoxylin-eosin (HE), PAS and Masson staining. Positive staining for hemoglobin confirmed the presence of hemoglobin casts (×200 and ×400, respectively).

**Final diagnosis:** 1 Acute kidney injury—hemoglobin cast nephropathy (biopsy-proven); 2. Complement-positive autoimmune hemolytic anemia (immune-mediated hemolysis of uncertain subtype).

Given the isolated C3-postive DAT, the preceding infectious-like illness, and the intravascular hemolysis, paroxysmal cold hemoglobinuria-a subtype of cold-antibody-mediated autoimmune hemolytic anemia (cAIHA)-is the clinically favored diagnosis, although it could not be confirmed because the Donath-Landsteiner test was unavailable.

## Follow-up and outcomes

Hospital day 10 (August 18): pre-dialysis Scr 279 μmol/L; 24 h urine output 1750 mL. The patient was discharged on day 21 (August 29, 2025) with improved symptoms, declining creatinine, and rising hemoglobin. Creatinine declined to 83 μmol/L by day 18 and 57 μmol/L by October 10, 2025, and remained stable at 51–57 μmol/L through the most recent follow-up on July 24, 2026 (approximately 11.5 months). Hemoglobin recovered to 125 g/L by October 10, 2025 and remained at 127–130 g/L thereafter, and Coombs tests were negative, including the anti-C3d antibody test. There was no relapse of hemolysis and no recurrence of renal dysfunction (Figure 2, 3).

**Figure 2:**
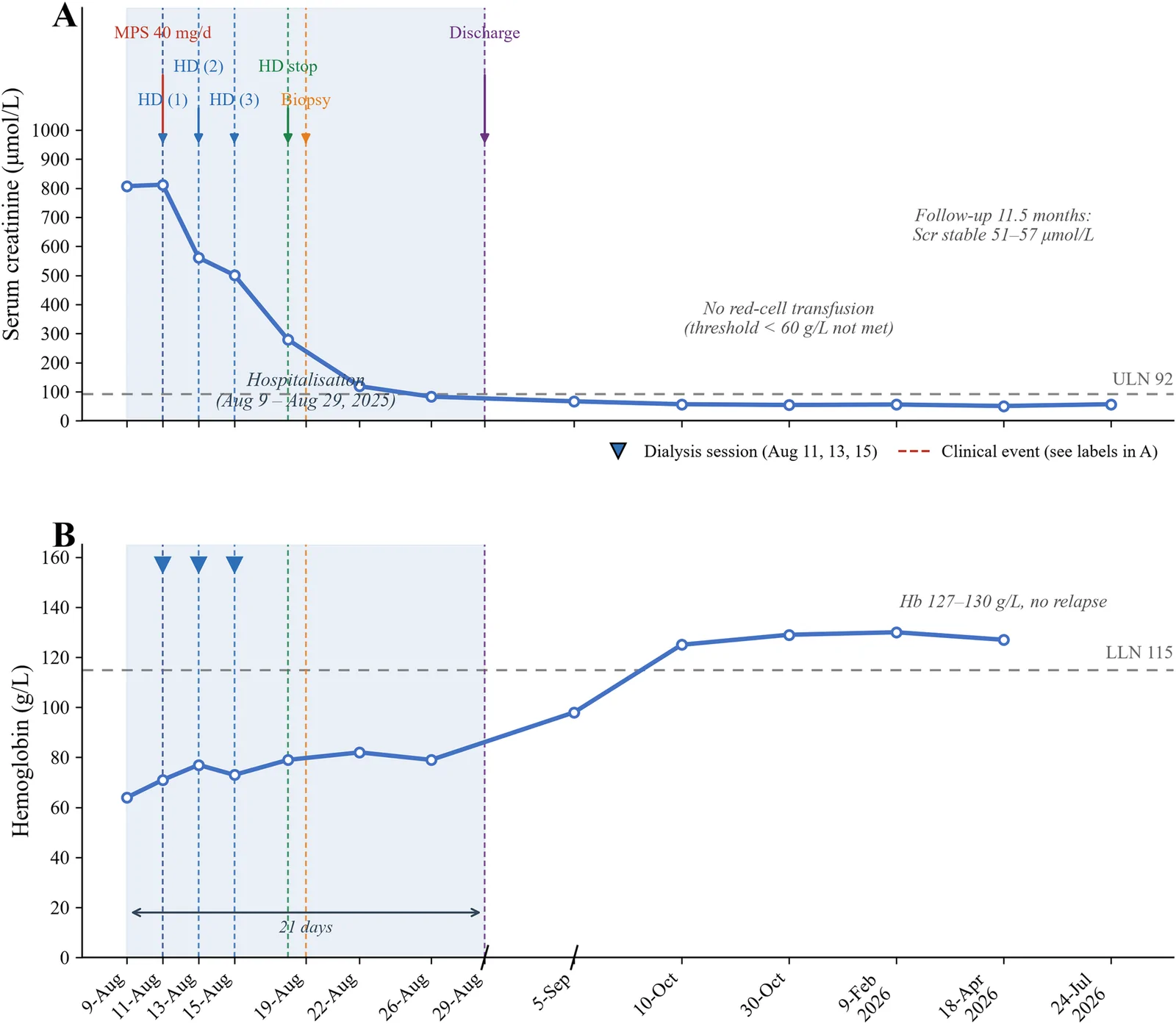
Trends in serum creatinine **(A)** and hemoglobin **(B)**. The patient presented with an initial serum creatinine of 807 μmol/L (upper limit of normal 92 μmol/L) and hemoglobin of 64 g/L (lower limit of normal 115 g/L) on hospital day 1. Annotated clinical timepoints mark each dialysis session (August 11, 13, and 15), initiation of methylprednisolone (August 11), renal biopsy (August 19), discontinuation of dialysis, and hospital discharge (August 29). No red-cell transfusion was administered. Serum creatinine declined progressively to 83 μmol/L by day 18 and remained stable at 51–57 μmol/L through July 24, 2026; hemoglobin recovered to the normal range within 45 days of discharge.

**Figure 3:**
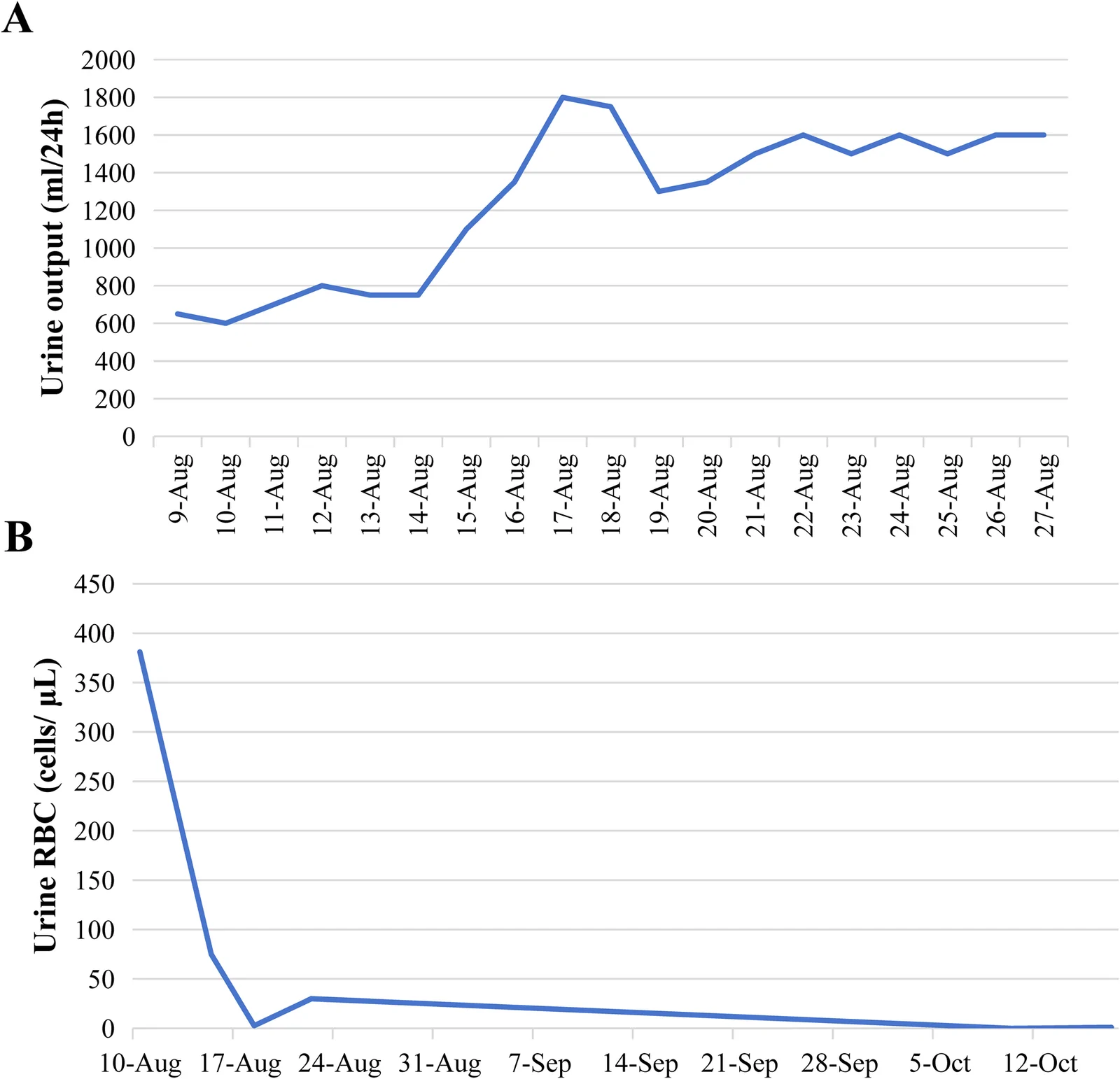
Trends in urine volume **(A)** and urine red blood cell count **(B)** urine volume recovered to normal by hospital day 10, and erythrocytes were no longer detected on urinalysis during follow-up after discharge.

## Discussion

Intravascular hemolysis is a relatively rare and life-threatening pathological process characterized by the destruction of red blood cells with release of free hemoglobin, and it is associated witha mortality rate as high as 11% (5). The most common disease associated with hemolysis is autoimmune hemolytic anemia (AIHA), an acquired hemolytic disorder with an incidence of 1–3 per 100,000 per year (6). Warm-antibody AIHA is the major subtype of autoimmune-mediated hemolytic anemia, and approximately half of cases are idiopathic. The clinical manifestations of AIHA are variable, including dyspnea, fatigue, headache, muscle weakness, pallor, and jaundice, ranging from mild compensated hemolysis to life-threatening severe disease. Because of the rarity, heterogeneity, and insufficient understanding of severe AIHA, the identification and management of severe cases are particularly complex. Das et al. and Ray et al. (7) defined “severe hemolysis” as meeting the following four criteria: hemoglobin <9 g/dL, indirect bilirubin >2 mg/dL, reticulocyte count >2%, and lactate dehydrogenase (LDH) > 500 IU/L; cases below these thresholds are classified as “moderate hemolysis”. In primary AIHA, the risk of mortality has been associated with Evans syndrome, infection, renal failure, and multiple treatments, and a hemoglobin level below 8 g/dL at onset is an important risk factor for intensive-care admission, recurrence, and death (7, 8).

The patient in this case was a late middle-aged female with an acute onset, presenting with transient bloody urine, edema, proteinuria, decreased urine output, and rapidly progressive renal dysfunction. Renal histopathology demonstrated acute tubular injury with hemoglobin casts confirmed by immunostaining, and the final diagnosis was acute kidney injury due to hemoglobin cast nephropathy with complement-positive autoimmune hemolytic anemia. Notably, the hemolytic episode was fulminant but self-attenuating: LDH peaked at 1,627 U/L with indirect hyperbilirubinemia one week before admission but had substantially fallen by admission. Although LDH had declined to 352 U/L on admission, it remained above the upper limit of normal, indicating persistent low-grade hemolysis. Together with renal anemia from acute kidney injury and reduced oral intake, this explains the continued decline in hemoglobin despite the improving acute hemolytic markers. Haptoglobin remained within the normal range despite hemolysis, which we attribute to its up-regulation as an acute-phase reactant during the concurrent inflammatory state; its subsequent downward drift as inflammation subsided is consistent with this explanation. Additional contributors to the progressive anemia included renal anemia and reduced oral intake, with no evidence of significant blood loss, hemodilution, phlebotomy, or dialysis-related losses.

The diagnostic attribution deserves particular care. The biopsy and hemoglobin immunostaining establish hemoglobin cast nephropathy- that is, exposure of the kidney to intravascular hemolysis- but they do not by themselves establish that the hemolysis was of autoimmune origin. The attribution rests on the serological evidence (an isolated C3-positively DAT with negative IgG). As the isolated complement positivity is more suggestive of cold-antibody-mediated hemolysis or paroxysmal cold hemoglobinuria, and because the Donath–Landsteiner test was unavailable, we designate the final diagnosis as complement-positive autoimmune hemolytic anemia (immune-mediated hemolysis of uncertain subtype) rather than warm-type AIHA. The coexistence of true (non-glomerular) hematuria does not exclude hemoglobin cast nephropathy but underscores the importance of the direct documentation provided by the positive hemoglobin immunostaining and the declining urine hemoglobin.

A review of the literature shows that hemoglobin cast nephropathy may result from endogenous and exogenous red-cell defects [AIHA, ABO incompatibility, paroxysmal nocturnal hemoglobinuria (PNH), sickle cell anemia, and Evans syndrome], drug (rifampicin, trimethoprim-sulfamethoxazole, vancomycin, bevacizumab), infectious (e.g., Mycoplasma), mechanical factors (cardiopulmonary bypass, prosthetic heart valves), and other factors such as sodium nitrite ingestion and disseminated intravascular coagulation (DIC) (9). A 2019 retrospective analysis of 27 cases of hemoglobin cast nephropathy by Arkana Laboratories in the United States found that the top three etiologies were AIHA (30%), drug-related factors (26%), and PNH (7%), accounting for 0.063% of native kidney biopsies-far less than the 0.78% for myoglobin cast nephropathy over the same period (9). A retrospective analysis of 56 cases from India (10) found that the leading causes to be rifampicin exposure (30.3%), snakebites (28.4%) and autoimmune hemolytic anemia (7.2%); these discrepancies likely reflect regional and socioeconomic differences. In the present case, screening tests for PNH, cold agglutination, and glucose-6-phosphate dehydrogenase (G6PD) deficiency werenegative, and the patient had no relevant drug, food, or surgical exposure. We therefore attribute the hemolysis to an autoimmune process, designated complement-positive AIHA of uncertain subtype.

The acute renal injury in this case shares a common pathogenesis with other etiologies of AKI: free hemoglobin released into plasma saturates haptoglobin; excess free hemoglobin dissociates from tetramers into dimmers, which chelate nitric oxide, pass the glomerular filtration barrier, and deposit in the renal tubules (9, 11, 12). After reabsorption by renal tubular epithelial cells, hemoglobin dissociates into heme and globin, and elevated intracellular heme induces tubular epithelial injury. The negative myoglobin immunostaining on biopsy, together with the positive hemoglobin staining, confirms that the casts were compased of hemoglobin rather than myoglobin.

The timing of corticosteroid therapy merits a cautious interpretation. Because the major hemolytic markers had already improved before corticosteroid initiation, we do not attribute hematologic recovery solely to corticosteroid responsiveness; improvement likely reflects both spontaneous attenuation of the fulminant hemolysis and a possible therapeutic effect of corticosteroids. At discharge the patient was advised to avoid cold exposure, to seek prompt evaluation of any febrile or infectious illness, and to undergo regular hematological and renal follow-up, given the possibility of recurrence after subsequent infections; no recurrence has occurred during 11.5 months of follow-up.

In summary, hemoglobin cast nephropathy is a rare clinical entity, and acute kidney injury induced by hemoglobin casts is extremely life-threatening despite sharing similar clinical features with AKI of other etiologies. This case provides the following clinical insights: when AKI and hemolytic anemia occur concomitantly, hemoglobin cast nephropathy should be strongly suspected; renal biopsy is valuable when the diagnosis or differential diagnosis remains uncertain, but is not mandatory in every clinically evident case of pigment nephropathy; and treatment primarily targets the underlying cause of hemolysis and mitigation of renal injury induced by tubular cast formation from red-cell components.

## Limitations

The diagnostic subtype of AIHA could not be fully resolved because monospecific IgA/IgM testing, eluate testing, and the Donath-Landsteiner test were unavailable in our institution. No microbiological or serological investigations (including Mycoplasma pneumoniae, EBV, and CMV) were performed, so the presumed infectious trigger remains unproven. A complete panel of intravascular-hemolysis biomarkers (including plasma free hemoglobin and urine hemosiderin) was not available, although the negative myoglobin stain and the absence of tubular hemosiderin deposition provided useful negative evidence. Stool Shiga-toxin and ADAMTS13 testing were not performed, although thrombotic microangiopathy was excluded on clinical and pathological grounds.

## Data Availability

The original contributions presented in the study are included in the article/Supplementary Material, further inquiries can be directed to the corresponding author/s.
